# In Silico Functional Networks Identified in Fish Nucleated Red Blood Cells by Means of Transcriptomic and Proteomic Profiling

**DOI:** 10.3390/genes9040202

**Published:** 2018-04-09

**Authors:** Sara Puente-Marin, Iván Nombela, Sergio Ciordia, María Carmen Mena, Verónica Chico, Julio Coll, María del Mar Ortega-Villaizan

**Affiliations:** 1 Instituto de Biología Molecular y Celular, Universidad Miguel Hernández, 03202 Elche, Spain; spuente@umh.es (S.P.-M.); inombela@umh.es (I.N.); vchico@umh.es (V.C.); 2 Unidad de Proteómica, Centro Nacional de Biotecnología (CNB-CSIC), 28049 Madrid, Spain; sciordia@cnb.csic.es (S.C.); mcmena@cnb.csic.es (M.C.M.); 3 Instituto Nacional de Investigación y Tecnología Agraria y Alimentaria (INIA), 28040 Madrid, Spain; juliocoll@inia.es

**Keywords:** rainbow trout, red blood cells, RNA-seq, de novo assembly, transcriptome, peptide fractionation, LC ESI-MSMS, proteome, functional network, immune response

## Abstract

Nucleated red blood cells (RBCs) of fish have, in the last decade, been implicated in several immune-related functions, such as antiviral response, phagocytosis or cytokine-mediated signaling. RNA-sequencing (RNA-seq) and label-free shotgun proteomic analyses were carried out for in silico functional pathway profiling of rainbow trout RBCs. For RNA-seq, a de novo assembly was conducted, in order to create a transcriptome database for RBCs. For proteome profiling, we developed a proteomic method that combined: (a) fractionation into cytosolic and membrane fractions, (b) hemoglobin removal of the cytosolic fraction, (c) protein digestion, and (d) a novel step with pH reversed-phase peptide fractionation and final Liquid Chromatography Electrospray Ionization Tandem Mass Spectrometric (LC ESI-MS/MS) analysis of each fraction. Combined transcriptome- and proteome- sequencing data identified, in silico, novel and striking immune functional networks for rainbow trout nucleated RBCs, which are mainly linked to innate and adaptive immunity. Functional pathways related to regulation of hematopoietic cell differentiation, antigen presentation via major histocompatibility complex class II (MHCII), leukocyte differentiation and regulation of leukocyte activation were identified. These preliminary findings further implicate nucleated RBCs in immune function, such as antigen presentation and leukocyte activation.

## 1. Introduction

Red blood cells (RBCs) are the most copious cell type in blood circulation and are well-known for their roles in respiration. Also, other roles such as modulation of angiogenesis, coagulation, vascular tone and inflammation have been described for mammalian RBCs (reviewed in Akbari A. 2011) [1]. In mammals, mature RBCs lack cell nuclei, organelles, and ribosomes [2]. In contrast, in non-mammalian vertebrates, RBCs have cell nuclei and organelles in their cytoplasm [3]. The role of nucleated RBCs as immune response intermediaries is a novel field of research [4]. RBCs, rich in hemoglobin, were thought to drive processes of gas exchange to tissues. However, in the recent past, a set of biological processes related to immunity, such as phagocytosis and presentation [5], interferons production [6,7,8,9] and cytokines production [7,8,10], have been reported in nucleated RBCs from different non-mammal vertebrate species. During the last decade, transcriptomic and proteomic sequencing have allowed us to identify many more genes and proteins in RBCs. Transcriptome sequencing of nucleated RBCs has identified the genes responsible for the expression of a wide spectrum of biological processes, including immune response [6,11]. On the other hand, proteomics sequencing of non-nucleated RBCs has significantly evolved [12,13,14,15], allowing us to significantly increase the number of identified proteins from a few hundred to almost 2700 proteins [12,13]. However, to our knowledge, no study on nucleated RBCs proteome sequencing has been published (although extensive research exists on cell proteome of the different cell types during human erythroid differentiation [16]).

**Figure 1:**
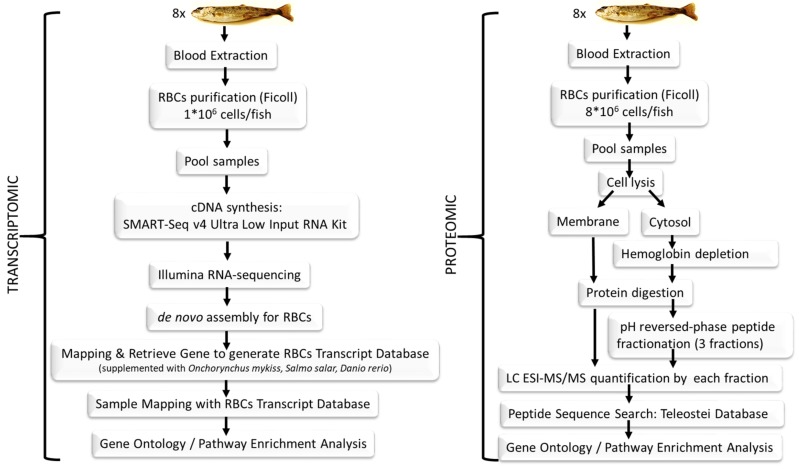
Schema representing the different steps in the experiment described here, from sample collection to data analysis.

In this manuscript we show a combined transcriptomic and proteomic evaluation of rainbow trout nucleated RBCs (see Figure 1 for a representative schema of the procedure). In order to achieve this, we performed RNA-sequencing (RNA-seq) and label-free shotgun proteomic analyses of RBCs pooled from eight fishes. For transcriptome profiling, a de novo assembly of rainbow trout RBCs was conducted to create a transcriptome database for RBCs gene mapping. For proteome profiling, we developed a novel proteomic analysis method that combined: (a) fractionation into cytosolic and membrane fractions, (b) hemoglobin removal of the cytosolic fraction, (c) protein digestion, and (d) a novel step with pH reversed-phase peptide fractionation and final Liquid Chromatography Electrospray Ionization Tandem Mass Spectrometric (LC ESI-MS/MS) analysis of each of the fractions.

In silico functional profiling revealed the presence of novel and striking networks for rainbow trout nucleated RBCs, mainly related to innate and adaptive immunity. Functional pathways related to regulation of hematopoietic cell differentiation, antigen presentation via major histocompatibility complex class II (MHCII), leukocyte differentiation, and regulation of leukocyte activation were found in rainbow trout RBCs transcriptome. This study provides new knowledge on the immune functions of nucleated RBCs.

## 2. Materials and Methods

### 2.1. Fish

Rainbow trout (*Oncorhynchus mykiss*) of approximately 6 cm, obtained from a commercial fish farm (PISZOLLA S.L., CIMBALLA FISH FARM, Zaragoza, Spain), were maintained at University Miguel Hernandez (UMH) facilities at 14 °C, with a re-circulating dechlorinated-water system, at a stocking density of 1 fish/3 L, and fed daily with a commercial diet (Skretting, Burgos, Spain). Fish were acclimatized to laboratory conditions over 2 weeks. All activities involving animal handling and animal care were done in accordance with EU Directive EC86/609.

### 2.2. Blood Sampling and Red Blood Cells Purification

Rainbow trout RBCs were obtained from the peripheral blood of fish which died through overexposure to tricaine (tricaine methanesulfonate, Sigma-Aldrich, Madrid, Spain; 0.2 g/L), as previously described [8]. Briefly, peripheral blood was sampled from the caudal vein. Then, RBCs were purified by two consecutive density gradient centrifugations (7206 *g*, Ficoll 1.007; Sigma-Aldrich). Purity of RBCs of 99.9% was estimated by optical microscopy evaluation (Figure S1). 

### 2.3. Transcriptome Sequencing

#### 2.3.1. Complementary DNA Library Preparation and Illumina Sequencing

RBCs isolated from eight fishes (10^6^ cells per fish) were lysed with 9.5 µL of 10× Lysis buffer (Clontech, Takara Bio, Mountain View, CA, USA) and 0.5 µL of ribonuclease (RNase) Inhibitor (Invitrogen, Thermo-Fischer Scientific Inc., Waltham, MA, USA), and preserved at −80 °C, until complementary DNA (cDNA) library construction.

Lysed RBCs from the eight fishes were pooled, and cDNA was directly produced from lysed cells using a SMART-Seq v4 Ultra Low Input RNA Kit (Clontech, Takara Bio). cDNA integrity was tested using a Bioanalyzer 2100 (Agilent, Santa Clara, CA, USA). The library construction was carried out using an Illumina Nextera XT Library Preparation Kit (Illumina Inc., San Diego, CA, USA). Generated cDNA fragments were sequenced with the lllumina Hiseq 2500 platform, using 100 bp paired-end sequencing reads. Sequence reads are available at SRA-NCBI, SRA-NCBI Accession SRP133501. RNA-Seq library preparation and sequencing were carried out by STABVida Lda (Caparica, Portugal).

#### 2.3.2. De Novo Assembly Bioinformatics Procedure

In order to create a transcript database specific to, or enriched for, rainbow trout RBCs, a de novo assembly of RBCs expressed short reads was carried out. CLC Genomics Workbench (version 9.5.4) [17] was used for expressed short reads de novo assembly. Raw data were filtered by removing short, duplicated and low quality reads. For each original read, the regions of the sequence to be removed were determined independently for each type of trimming operation: Quality trimming (based on quality ratings), and Ambiguity trimming. The trimming parameters applied were: ambiguous limit = 2 nucleotides, quality limit = 0.01 (error probability), minimum number of nucleotides = 30. After quality trimming, raw sequence data were de novo assembled. One list of sequences corresponding to generated contigs, and one mapping file were generated. After initial contig creation, reads were mapped back to contigs for assembly correction, using the following parameters: word size = 54, bubble size = 50, length fraction = 0.8 and similarity fraction = 0.8. To remove redundancy from assemblies, generated contigs were analyzed with CD HIT EST (Version 4.6) [18,19], using the following parameters: -c 0.85 -n 8.

#### 2.3.3. BLASTing of Assembled Contigs, Gene Sequence Retrieval, Red Blood Cells Transcript Database Construction and Functional Annotation

De novo assembled contigs were BLASTed (using Nucleotide Basic Local Alignment Search Tool, BLASTn, https://blast.ncbi.nlm.nih.gov/Blast.cgi), with a cut off E-value of 1.00 × 10^−3^) against a local Teleostei messenger RNA (mRNA) Reference Sequence (RefSeq) database downloaded from NCBI (https://www.ncbi.nlm.nih.gov, last update: 20072017), using Blast2GO PRO version 4.1.9 [20]. Then, full sequences of the top blast hit obtained in the previous step were retrieved from NCBI, based on accession ids. Duplicated and similar sequences with 95% similarity were removed. These steps were performed using Blast2GO PRO version 4.1.9. Resulting mRNA RefSeq curated database{Ortega-Villaizan, 2018 #28} (referred to hereafter as RBCs transcript database [21]), was enriched with rainbow trout (NCBI, last update: 09082017), Atlantic salmon-*Salmo salar*-(NCBI, last update: 09082917), and zebrafish-*Danio rerio*-(NCBI, last update: 31072017) mRNA RefSeq annotations in NCBI. This curated and enriched database was used as a reference for following sample mapping and annotation analyses. The RBCs transcript database was finally annotated against local Teleostei protein RefSeq database downloaded from NCBI (last update: 17072017), using Basic Local Alignment Search Tool (BLASTx, https://blast.ncbi.nlm.nih.gov/Blast.cgi) with a cut off E value of 1.00 × 10^−3^. 

#### 2.3.4. Red Blood Cells Transcriptome Mapping and Gene Expression Profiling

Sample raw sequence data was mapped against the RBCs transcript database using CLC Genomics Workbench (version 10.1.1) [22]. Before mapping, analysis started with the trimming of raw sequences to generate high quality data only. For each original read, the regions of the sequence to be removed were determined independently for each type of trimming operation: Quality trimming (based on quality ratings), Ambiguity trimming, and Length trimming. The following trimming parameters were applied: ambiguous limit = 2 nucleotides, quality limit = 0.01 (error probability), minimum number of nucleotides = 15. High quality sequencing reads (approximately 40 million reads) were mapped against RBCs transcript database, using the following parameters: length fraction = 0.6, similarity fraction = 0.5. Gene prediction and annotation was conducted on the RBCs transcripts database. The expression level of genes was obtained by counting the number of reads mapped to a gene. 

### 2.4. Proteome Sequencing

#### 2.4.1. Protein Digestion

RBCs isolated from eight fishes (8 × 10^6^ cells per fish) were pooled and pelleted by centrifugation (5 min, 700× *g*). Supernatant was removed, and cell pellet (~70–100 μL) was mixed with 250 μL of deionized water, and then frozen at −80 °C for 3 h. After thawing, it was centrifuged at 17,000× *g* for 20 min at 4 °C, to separate cytosolic supernatant and pelleted membrane fractions. To purify the membrane fraction, the pellet was washed twice with 500 µL of deionized water, and then centrifuged at 20,000× *g* for 10 min at 4 °C. After washing, the new membrane pellet was dissolved with 200 µL of chaotropic lysis buffer containing 8.4 M urea (USB Corporation, Cleveland, OH, USA), 2.4 M thiourea (Sigma-Aldrich), 5% CHAPS (Sigma-Aldrich), 5 mM TCEP (Sigma-Aldrich) and a protease inhibitor cocktail (Sigma-Aldrich), for 15 min, on ice. Homogenization of the membrane pellet was achieved by ultrasonication for 5 min on ultrasonic bath Branson 2510 (Marshall Scientific, Hampton, NH, USA). The sonicated membrane was centrifuged again at 20,000× *g* for 10 min at 4 °C, and the supernatant containing solubilized membrane fraction proteins was used for further analysis. Then, 40 µg of protein was precipitated using the methanol/chloroform method [23], and re-suspended in 20 µL of multichaotropic sample solution composed of 7 M Urea, 2 M thiourea and 10 mM triethylammonium bicarbonate (TEAB) (Sigma-Aldrich), called hereafter UTT buffer.

The cytosolic fraction, approximately 300 μL, was depleted of hemoglobin using HemoVoid^TM^ kit (Biotech Support Group, Monmouth Junction, NJ, USA), in accordance with the manufacturer’s instructions [24]. After hemoglobin removal, the eluted fraction was transferred to a Pall Omega Nanosep^®^ (Pall Corporation, Port Washington, NY, USA) centrifugal filter device (molecular weight cut-off (MWCO) 10 kDa), and concentrated by centrifugation at 14,000× *g* for 15 min at 4 °C. Finally, the hemoglobin depleted-cytosolic fraction were dialyzed with 300 µL of UTT buffer, and concentrated into a volume of ~50–80 µL (20 min, 14,000× *g*, 4 °C). Then, 40 µg of protein were diluted with 20 µL of multichaotropic sample solution UTT buffer.

Both protein fractions were reduced with 2 µL of 50 mM TCEP, pH 8.0, at 37 °C for 60 min, before 1 µL of 200 mM cysteine-blocking reagent MMTS (SCIEX, Foster City, CA, USA) was added for 10 min at room temperature. Then, the cytosolic and membrane fractions were diluted to 140 µL with 25 mM TEAB, to reduce the urea concentration. Finally, digestions were initiated by adding 6 and 2 µg respectively of Pierce MS-grade trypsin (Thermo-Fisher Scientific Inc., Waltham, MA, USA) to each fraction, in a ratio of 1:20 (*w*/*w*), and then incubated at 37 °C overnight on a shaker. The fraction digestions were evaporated to dryness in a vacuum concentrator.

#### 2.4.2. pH Reversed-Phase Peptide Fractionation

Offline high pH reversed-phase peptide fractionation of peptides from cytosolic fraction was performed on a SmartLine (Knauer, Berlin, Germany) high pressure liquid chromatography (HPLC) system using an XBridge C18 column (100 × 2.1 mm, 5 μm particle) (Waters Corporation, Milford, MA, USA). Mobile phases A and B were used for chromatography. The composition of mobile phase A was 10 mM ammonium hydroxide (pH 9.4) (Sigma-Aldrich), whereas composition of mobile phase B was 80% methanol (Scharlab S.L, Barcelona, Spain) and 10 mM ammonium hydroxide (pH 9.3). Dried-up peptide pellet was dissolved in 100 μL of mobile phase A, injected into a sample loop, and then fractionated using a linear gradient of 0–100% mobile phase B at 150 μL/min for 90 min. Thirty fractions were collected and then pooled, with an alternating numerical sequence, into three fractions (i.e., fractions 1 + 4 + 7 + 10 + 13 + 16 + 19 + 22 + 25 + 28) and dried.

#### 2.4.3. Liquid Chromatography and Mass Spectrometry Analysis

Peptide fractions were cleaned/desalted using Stage-Tips with Empore 3M C18 disks (Sigma-Aldrich). One microgram of each peptide fraction was used for a 1D-nano LC ESI-MS/MS analysis, using a nano-liquid chromatography system (Eksigent Technologies nano LC Ultra 1D plus; SCIEX, Foster City, CA, USA), coupled to a high speed Triple TOF 5600 mass spectrometer (SCIEX) with a Nanospray III source. The analytical column used was a silica-based reversed phase Acquity UPLC^®^ M-Class Peptide BEH C18 Column (Waters Corporation). The trap column was a C18 Acclaim PepMap^TM^ 100 (Thermo-Fisher Scientific Inc.), 100 µm × 2 cm, 5 µm particle diameter, 100 Å pore size, switched on-line with the analytical column. A loading pump delivered a solution of 0.1% formic acid in water at 2 µL/min. The nano-pump provided a flow-rate of 250 nL/min, and was operated under gradient elution conditions. Cytosolic peptide fractions were separated using a 150 min gradient ranging from 2% to 90% mobile phase B (mobile phase A: 2% acetonitrile (Scharlab S.L), 0.1% formic acid (Sigma-Aldrich); mobile phase B: 100% acetonitrile, 0.1% formic acid). Two hundred & fifty minutes’ gradient was used for the membrane fraction, using the same gradient conditions. Injection volume was 5 µL. 

Data were acquired using an ionspray voltage floating 2300 V, curtain gas 35, interface heater temperature 150, ion source gas 125 and declustering potential 150 V. For Information-Dependent Acquisition (IDA) parameters, 0.25 s mass spectrometry (MS) survey scan in the mass range of 350–1250 Da were followed by 35 MS/MS scans of 100 ms in the mass range of 100–1800. Switching criteria were set to ions greater than mass-to-charge ratio (*m*/*z*) 350 and smaller than *m*/*z* 1250 with a charge state of 2–5 and an abundance threshold >90 counts (cps). Former target ions were excluded for 15 s.

#### 2.4.4. Proteomics Data Analysis and Sequence Search

Mass spectrometry data obtained were processed using PeakView 2.2 Software (SCIEX [25]) and exported as mgf files, which were then searched, using Mascot Server v2.5.1 (Matrix Science, London, UK), against a protein database including Teleostei protein sequences from Uniprot/Swissprot Knowledgebase (last update: 20170412, 2.542.118 sequences), together with commonly occurring contaminants. Search parameters were set as follows: enzyme, trypsin; allowed missed cleavages, 2; methylthiolation (C) as fixed modification; and acetyl (Protein N-term), Oxidation (M), Gln → pyro-Glu (N-term Q) and Glu → pyro-Glu (N-term E) as variable modifications. Peptide mass tolerance was set to ±25 ppm for precursors and 0.05 Da for fragment masses. The confidence interval for protein identification was set to ≥95% (*p* value < 0.05) and only peptides with an individual ion score above the 1% False Discovery Rate (FDR) at PSM (peptide-to-spectrum matches) level were considered to have been correctly identified.

### 2.5. Pathway Enrichment Analysis

In order to evaluate functionally grouped Gene Ontology (GO) and pathway annotation networks of expressed genes and proteins, pathway enrichment analysis was performed using the ClueGO [26] and CluePedia [27] Cytoscape [28] plugins. The GO Immune System Process functional pathway database was used. *p* value ≤ 0.05 and Kappa score of 0.4 served as threshold values. Protein-protein interaction (PPI) networks were analyzed using STRING v10.5 (http://string.embl.de/) [29], with a medium confidence score threshold of 0.4. The *Homo sapiens* model organism was used for ClueGO and STRING analyses. Gene symbols were obtained through sequence homology of RBCs transcript database genes with *Homo sapiens* orthologues, using Blast2GO version 4.1.9.

### 2.6. RNA Extraction and Reverse Transcription Real Time Polymerase Chain Reaction Analysis

RNA extraction and reverse transcription real time polymerase chain reaction (RT-qPCR) was performed as previously described [8], using specific primers and probe for *mhcII* gene (Forward:TGCCATGCTGATGTGCAG; Reverse: GTCCCTCAGCCAGGTCACT; Probe: CGCCTATGACTTCTACCCCAAACAAAT) [30]. Gene expression was analyzed by the 2^−ΔCt^ method [31] and *ef1α* gene (Forward: ACCCTCCTCTTGGTCGTTTC; Reverse: TGATGACACCAACAGCAACA; Probe: GCTGTGCGTGACATGAGGCA) [32] was used as endogenous control.

Besides, RNA extracted from RTS-11 cell line [33] (donated by Dr. Niels Bols) and RTG-2 cell line (ATCC^®^ CCL-55™) were respectively used as antigen presenting cell (APC) and non-APC cell types, for *mhcII* gene expression comparison.

### 2.7. RBCs Single-Cell Sorting

RBCs from one fish were single-cell sorted, in order to obtain a sample of pure RBCs (20–30 cells), using BD FACSJazz™ cell sorter (BD Biosciences, Madrid, Spain). Sorted RBCs were visualized using an IN Cell Analyzer 6000 (GE Healthcare, Little Chalfont, UK) cell imaging system. The sample was lysed with 9.5 µL of 10× Lysis buffer and 0.5 µL of RNase Inhibitor, and preserved at −80 °C until cDNA library construction.

## 3. Results and Discussion

**Figure 2:**
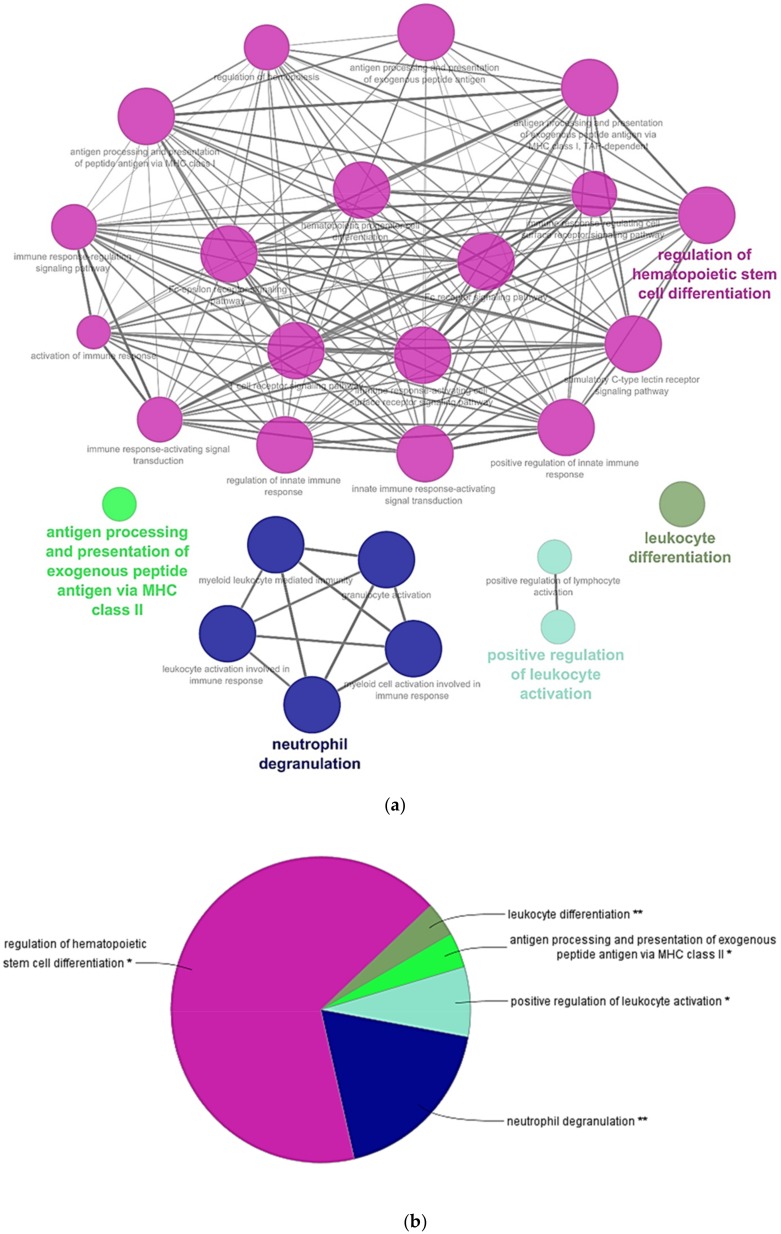
Cytoscape pathway network of significantly over-represented Immune System Process Gene Ontology (GO)-terms in RBCs transcriptome and proteome profiling common genes and proteins. (**a**) Pathway network. Each node represents a GO-term from Immune System Process. Node size shows GO-term significance (*p* value): smaller *p* value, larger node size. Edge (lines) between nodes indicate the presence of common genes: thicker line implies a larger overlap. GO-terms are classified into several function groups (different node color). The label of the most significant GO-term for each group is highlighted. (**b**) A pie chart of Immune System Process function groups. Asterisks denote GO-term significance. Functional groups are labelled as follows: Dark pink = regulation of hematopoietic stem cell differentiation, dark blue = neutrophil degranulation, light blue = positive regulation of leukocyte activation, light green = antigen processing and presentation of exogenous peptide antigen via MHCII, and dark green = leukocyte differentiation. A list of all over-represented terms and statistics is provided in Table S2.

**Figure 3:**
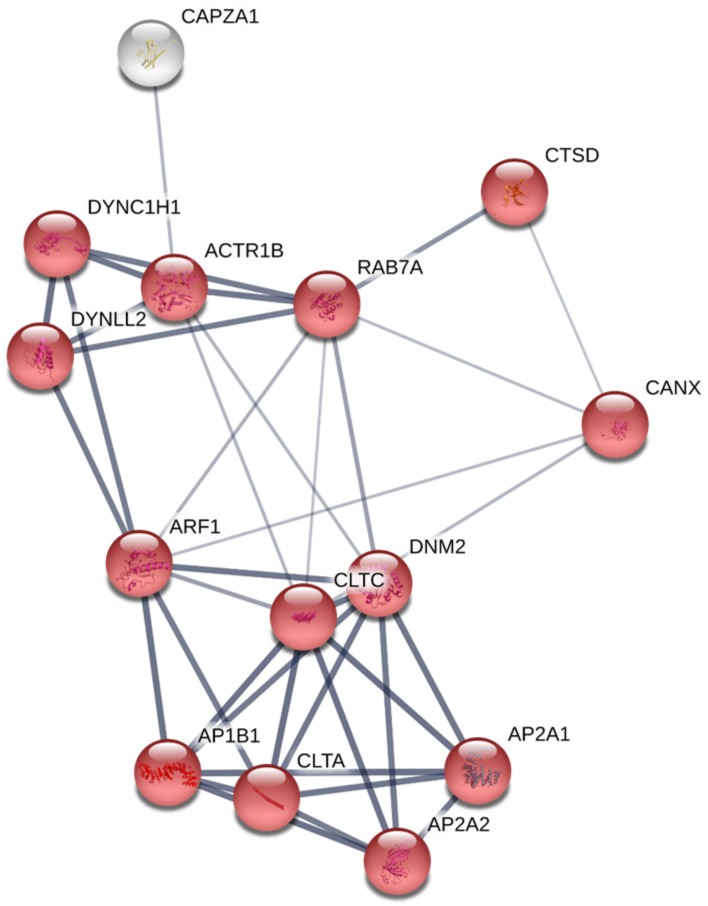
Constructed protein-protein interactions of a set of proteins of antigen processing and presentation of exogenous peptide antigen via MHCII GO-term using STRING software. Nodes represent proteins, while edges denote the interactions between two proteins. Red nodes highlight proteins functionally annotated with STRING software in GO-term antigen processing and presentation of exogenous peptide antigen via MHCII. White nodes represent proteins not functionally annotated in the highlighted GO-term. Network edge line thickness indicates the strength of data support.

RBCs transcriptome profiling identified 14,008 genes, from which 13,937 genes were considered expressed since they were detected above a threshold of 10 reads mapped. Table 1 shows statistics of de novo assembly, RNA-seq raw data and mapping. Conversely, proteome profiling identified 1.770 proteins, where 724 proteins had more than 2 PSMs. Among those genes with more than 10 gene reads and proteins with more than 2 PSMs and 670 genes and proteins (Table S1), were common to both transcriptome and proteome profiling respectively (Figure S2). A Cytoscape pathway enrichment analysis, with Immune System Process GO-terms, was performed in order to evaluate functionally grouped GO-terms and pathway annotation networks which are mainly represented in rainbow trout nucleated RBCs immune response. Results showed five strongly represented networks of interest: (i) regulation of hematopoietic stem cell differentiation, (ii) neutrophil degranulation, (iii) positive regulation of leukocyte activation, (iv) antigen processing and presentation of exogenous peptide antigen via MHCII, and (v) leukocyte differentiation (Figure 2a,b, Table S2). Subsequently, an interactome network was built for each GO-term set of proteins, to identify protein-protein interactions, and predict functional associations. We found that proteins grouped in antigen processing and presentation of exogenous peptide antigen via MHCII network highly interacted with each other (Figure 3), with a FDR *p* value 1.37 × 10^−27^ and PPI enrichment *p* value < 1.0 × 10^−16^. Fourteen proteins identified in this GO-term were: ACTR1B (ARP1 actin related protein 1 homolog B), AP1B1 (adaptor related protein complex 1 beta 1 subunit), AP2A1 (adaptor related protein complex 2 alpha 1 subunit), AP2A2 (adaptor related protein complex 2 alpha 2 subunit), ARF1 (ADP ribosylation factor 1), CANX (calnexin), CAPZA1 (capping actin protein of muscle Z-line alpha subunit 1), CLTA (clathrin light chain A), CLTC (clathrin heavy chain), CTSD (cathepsin D), DNM2 (dynamin 2), DYNC1H1 (dynein cytoplasmic 1 heavy chain 1), DYNLL2 (dynein light chain LC8-type 2), RAB7A (member RAS oncogene family). Moreover, the expression of these genes was corroborated in a single-cell sorted RBCs RNA-seq. Gene reads are indicated in Table S3. Besides, MHCII gene reads were detectable in single-cell sorted RBCs RNA-seq (Table S3).

These proteins, among others, may provide nucleated RBCs with the essential machinery to participate in the production of antigenic peptides, and their loading onto MHCII molecules within the compartments of endosomal–lysosomal system [34]. Unlike MHCI molecule—which is widely expressed on the cell surface of nearly all nucleated cells, including nucleated RBCs [35]—MHCII molecules are generally restricted to some endothelial cells and a subset of antigen-presenting cells (APCs), such as macrophages, dendritic cells, and B cells [36]. To our knowledge, there is only one record describing low levels of transcripts expression for MHC II in chicken nucleated RBCs [37]. We have also observed transcripts expression in rainbow trout nucleated RBCs (Figure S3). Moreover, it has been described how rainbow trout nucleated RBCs were shown to engulf *Candida albicans*, and presented it to macrophages [5]. Taken altogether, this evidence strongly suggests that nucleated RBCs may participate in antigen presentation, via MHCII, as professional APCs. 

These findings have broad implications in the knowledge of nucleated RBCs immune functions, since they open a novel topic of investigation where nucleated RBCs may act as professional APCs, and may be participants of the immunological synapse of T- and NK-cells. The function of MHCII pathway molecules in nucleated RBCs, and their role under viral infection scenarios, remains to be studied, and constitutes a part of our ongoing research.

## Figures and Tables

**Table 1:** De novo assembly, RNA-sequencing (RNA-seq) raw data and mapping statistics.

**De Novo Assembly**	
Total reads	404,825,036
Number of aligned reads	286,555,140
Total contigs	1,056,546
Contigs after CD HIT EST c 0.85	862,667
**RBCs Transcript Database**	
Genes after assembly BLAST, gene retrieval, removal of duplicates and 95% similar sequences	106,361
Genes after adding *Oncorhynchus mykiss*, *Salmo salar*, and *Danio rerio* NCBI sequences	137,444
**Raw Data and Mapping**	
Total reads	93,177,954
Reads after trimming	92,391,474
Mapped reads	52,118,053
Un-mapped reads	40,273,421

## Supplementary Materials

The following are available online at http://www.mdpi.com/2073-4425/9/4/202/s1, Figure S1: Brightfield microscopy image of RBCs after two consecutive density gradient centrifugations, Figure S2: Venn diagram of proteomic and transcriptomic results, Figure S3: *mhcII* gene expression, by means of RT-qPCR, in rainbow trout nucleated RBCs, RTS-11 and RTG-2 cell types, relative to *ef1α* endogenous control, Table S1: Common genes and proteins related to Immune System Process GO-terms overrepresented in RBCs, Table S2: List of all over-represented Immune System Process GO-terms in RBCs transcriptome and proteome profiling common genes and proteins, Table S3: Sorted RBCs RNA-seq reads mapped to the set of genes of antigen processing and presentation of exogenous peptide antigen via MHC class II GO-term.
