# Supplementary material for: In Silico Functional Networks Identified in Fish Nucleated Red Blood Cells by Means of Transcriptomic and Proteomic Profiling
Source: Genes (Basel). 2018 Apr 9;9(4):202. doi: 10.3390/genes9040202 (PMC5924544; doi:10.3390/genes9040202)
Supplement: Supplementary file 1 [file genes-09-00202-s001.zip › genes-279288_Table S1.docx]

**Table S1.** Common genes and proteins related to Immune System Process GO-terms overrepresented in RBCs. Protein symbol, description, RNA-seq gene reads and protein PSMs (peptide-to-spectrum matches)

| Gene Symbol | Description | Gene reads | PSMs |
| --- | --- | --- | --- |
| GDI2 | GDP dissociation inhibitor 2 | 53348 | 103 |
| FLOT2 | flotillin 2 | 29509 | 9 |
| MYH9 | myosin heavy chain 9 | 23991 | 5 |
| STAT1 | signal transducer and activator of transcription 1 | 21714 | 5 |
| STOM | stomatin | 13006 | 3 |
| ALDOA | aldolase, fructose-bisphosphate A | 8056 | 3 |
| LGALS9 | galectin 9 | 4724 | 12 |
| ILF2 | interleukin enhancer binding factor 2 | 4137 | 9 |
| RAP2B | RAP2B, member of RAS oncogene family | 3448 | 33 |
| CAPN1 | calpain 1 | 3446 | 12 |
| HSPA1A | heat shock protein family A (Hsp70) member 1A | 3160 | 5 |
| CALM1 | calmodulin 1 | 2952 | 40 |
| EZR | ezrin | 2913 | 29 |
| PAK1 | p21 (RAC1) activated kinase 1 | 1870 | 3 |
| TFRC | transferrin receptor | 1725 | 11 |
| CANX | calnexin | 1685 | 8 |
| PGM1 | phosphoglucomutase 1 | 1583 | 3 |
| EIF6 | eukaryotic translation initiation factor 6 | 1350 | 3 |
| ARPC5 | actin related protein 2/3 complex subunit 5 | 1329 | 4 |
| ERAP1 | endoplasmic reticulum aminopeptidase 1 | 1280 | 6 |
| SERPINB6 | serpin family B member 6 | 1272 | 7 |
| RAB10 | RAB10, member RAS oncogene family | 1253 | 6 |
| CAPZA1 | capping actin protein of muscle Z-line alpha subunit 1 | 1229 | 4 |
| A2M | alpha-2-macroglobulin | 1075 | 4 |
| ESCO2 | establishment of sister chromatid cohesion N-acetyltransferase 2 | 1053 | 25 |
| PRKACA | protein kinase cAMP-activated catalytic subunit alpha | 1053 | 6 |
| AP1B1 | adaptor related protein complex 1 beta 1 subunit | 1052 | 12 |
| ANPEP | alanyl aminopeptidase, membrane | 1049 | 11 |
| GBP1 | guanylate binding protein 1 | 1026 | 3 |
| UROD | uroporphyrinogen decarboxylase | 1019 | 7 |
| PPP3CA | protein phosphatase 3 catalytic subunit alpha | 1015 | 3 |
| HSP90B1 | heat shock protein 90 beta family member 1 | 966 | 6 |
| PSMC6 | proteasome 26S subunit, ATPase 6 | 956 | 21 |
| PSMB6 | proteasome subunit beta 6 | 949 | 4 |
| PAFAH1B1 | platelet activating factor acetylhydrolase 1b regulatory subunit 1 | 904 | 14 |
| APOB | apolipoprotein B | 884 | 3 |
| CLTC | clathrin heavy chain | 854 | 35 |
| DNM2 | dynamin 2 | 840 | 5 |
| CPNE1 | copine 1 | 804 | 22 |
| SERPINB1 | serpin family B member 1 | 741 | 26 |
| HUWE1 | HECT, UBA and WWE domain containing 1, E3 ubiquitin protein ligase | 714 | 6 |
| HSPA8 | heat shock protein family A (Hsp70) member 8 | 712 | 43 |
| PDXK | pyridoxal kinase | 694 | 5 |
| RAB5C | RAB5C, member RAS oncogene family | 668 | 3 |
| HDAC5 | histone deacetylase 5 | 576 | 3 |
| DYNLL2 | dynein light chain LC8-type 2 | 572 | 3 |
| UBE2D2 | ubiquitin conjugating enzyme E2 D2 | 567 | 3 |
| PA2G4 | proliferation-associated 2G4 | 564 | 4 |
| CLTA | clathrin light chain A | 550 | 3 |
| IDH1 | isocitrate dehydrogenase (NADP(+)) 1, cytosolic | 547 | 9 |
| RPS3 | ribosomal protein S3 | 490 | 14 |
| HSPA9 | heat shock protein family A (Hsp70) member 9 | 479 | 4 |
| NR1H4 | nuclear receptor subfamily 1 group H member 4 | 472 | 18 |
| PGAM1 | phosphoglycerate mutase 1 | 467 | 3 |
| CPNE3 | copine 3 | 465 | 7 |
| DDX3X | DEAD-box helicase 3, X-linked | 463 | 4 |
| CRK | CRK proto-oncogene, adaptor protein | 448 | 5 |
| ARF1 | ADP ribosylation factor 1 | 441 | 4 |
| ACTR3 | ARP3 actin related protein 3 homolog | 407 | 3 |
| PSMC3 | proteasome 26S subunit, ATPase 3 | 389 | 15 |
| FLOT1 | flotillin 1 | 384 | 8 |
| CTSD | cathepsin D | 378 | 6 |
| GPI | glucose-6-phosphate isomerase | 368 | 59 |
| AP2A1 | adaptor related protein complex 2 alpha 1 subunit | 356 | 6 |
| DYNC1H1 | dynein cytoplasmic 1 heavy chain 1 | 355 | 4 |
| HMGB2 | high mobility group box 2 | 352 | 82 |
| PURB | purine rich element binding protein B | 330 | 3 |
| UBB | ubiquitin B | 321 | 19 |
| IMPDH1 | inosine monophosphate dehydrogenase 1 | 312 | 3 |
| PHB | prohibitin | 287 | 3 |
| PSMB7 | proteasome subunit beta 7 | 286 | 5 |
| SPTAN1 | spectrin alpha, non-erythrocytic 1 | 283 | 190 |
| RAB7A | RAB7A, member RAS oncogene family | 282 | 6 |
| PRDX5 | peroxiredoxin 5 | 261 | 5 |
| SAMHD1 | SAM and HD domain containing deoxynucleoside triphosphate triphosphohydrolase 1 | 255 | 18 |
| SUMO1 | small ubiquitin-like modifier 1 | 252 | 3 |
| FADD | Fas associated via death domain | 243 | 6 |
| FRK | fyn related Src family tyrosine kinase | 234 | 3 |
| AP2A2 | adaptor related protein complex 2 alpha 2 subunit | 230 | 4 |
| MIF | macrophage migration inhibitory factor | 230 | 4 |
| PPIA | peptidylprolyl isomerase A | 228 | 31 |
| GLO1 | glyoxalase I | 222 | 6 |
| PLCG2 | phospholipase C gamma 2 | 221 | 3 |
| ALAD | aminolevulinate dehydratase | 214 | 184 |
| CREG1 | cellular repressor of E1A stimulated genes 1 | 214 | 29 |
| PAK3 | p21 (RAC1) activated kinase 3 | 212 | 3 |
| PSMA1 | proteasome subunit alpha 1 | 211 | 19 |
| VCP | valosin containing protein | 197 | 4 |
| CAND1 | cullin associated and neddylation dissociated 1 | 178 | 5 |
| HSP90AB1 | heat shock protein 90 alpha family class B member 1 | 177 | 36 |
| PSMA6 | proteasome subunit alpha 6 | 176 | 11 |
| LGALS1 | galectin 1 | 176 | 6 |
| PSME2 | proteasome activator subunit 2 | 161 | 12 |
| PSMD11 | proteasome 26S subunit, non-ATPase 11 | 157 | 20 |
| CCT8 | chaperonin containing TCP1 subunit 8 | 155 | 8 |
| ABCE1 | ATP binding cassette subfamily E member 1 | 153 | 3 |
| PSMD2 | proteasome 26S subunit, non-ATPase 2 | 149 | 18 |
| PSMD3 | proteasome 26S subunit, non-ATPase 3 | 148 | 14 |
| ACTG1 | actin gamma 1 | 146 | 107 |
| H3F3A | H3 histone family member 3A | 145 | 105 |
| ACTR1B | ARP1 actin related protein 1 homolog B | 145 | 4 |
| EEF2 | eukaryotic translation elongation factor 2 | 134 | 49 |
| PSMC2 | proteasome 26S subunit, ATPase 2 | 120 | 10 |
| PRKACB | protein kinase cAMP-activated catalytic subunit beta | 119 | 7 |
| UBC | ubiquitin C | 116 | 14 |
| PSMD1 | proteasome 26S subunit, non-ATPase 1 | 115 | 17 |
| PSMD12 | proteasome 26S subunit, non-ATPase 12 | 115 | 13 |
| MAN2B1 | mannosidase alpha class 2B member 1 | 114 | 4 |
| CCT2 | chaperonin containing TCP1 subunit 2 | 113 | 29 |
| LCP1 | lymphocyte cytosolic protein 1 | 111 | 5 |
| PSMD7 | proteasome 26S subunit, non-ATPase 7 | 109 | 6 |
| CAT | catalase | 103 | 117 |
| CA2 | carbonic anhydrase 2 | 102 | 154 |
| PKM | pyruvate kinase M1/2 | 98 | 6 |
| KPNB1 | karyopherin subunit beta 1 | 95 | 22 |
| ACTB | actin beta | 88 | 154 |
| UBE2N | ubiquitin conjugating enzyme E2 N | 87 | 3 |
| RPSA | ribosomal protein SA | 84 | 13 |
| PSMC1 | proteasome 26S subunit, ATPase 1 | 84 | 9 |
| KARS | lysyl-tRNA synthetase | 79 | 8 |
| PDIA3 | protein disulfide isomerase family A member 3 | 77 | 7 |
| PSMB5 | proteasome subunit beta 5 | 77 | 3 |
| TUBB2A | tubulin beta 2A class IIa | 76 | 21 |
| PSME1 | proteasome activator subunit 1 | 74 | 20 |
| NIT2 | nitrilase family member 2 | 72 | 11 |
| PSMA5 | proteasome subunit alpha 5 | 68 | 21 |
| PSMA3 | proteasome subunit alpha 3 | 68 | 8 |
| CASP8 | caspase 8 | 65 | 3 |
| CAP1 | cyclase associated actin cytoskeleton regulatory protein 1 | 62 | 12 |
| HSPD1 | heat shock protein family D (Hsp60) member 1 | 55 | 22 |
| PSMD8 | proteasome 26S subunit, non-ATPase 8 | 55 | 8 |
| PRDX6 | peroxiredoxin 6 | 52 | 10 |
| PSMB1 | proteasome subunit beta 1 | 51 | 10 |
| PSMD6 | proteasome 26S subunit, non-ATPase 6 | 51 | 9 |
| PSMD13 | proteasome 26S subunit, non-ATPase 13 | 50 | 11 |
| PRDX4 | peroxiredoxin 4 | 48 | 20 |
| PSMC5 | proteasome 26S subunit, ATPase 5 | 47 | 22 |
| PSMB9 | proteasome subunit beta 9 | 47 | 4 |
| RPS19 | ribosomal protein S19 | 47 | 3 |
| PSMA4 | proteasome subunit alpha 4 | 41 | 11 |
| PSMD14 | proteasome 26S subunit, non-ATPase 14 | 37 | 3 |
| PSMB3 | proteasome subunit beta 3 | 36 | 12 |
| PSMA2 | proteasome subunit alpha 2 | 35 | 14 |
| PSMC4 | proteasome 26S subunit, ATPase 4 | 35 | 7 |
| CSTB | cystatin B | 33 | 3 |
| PSMB4 | proteasome subunit beta 4 | 25 | 10 |
| PSMB2 | proteasome subunit beta 2 | 23 | 15 |
| EEF1A1 | eukaryotic translation elongation factor 1 alpha 1 | 22 | 50 |
| HEXB | hexosaminidase subunit beta | 22 | 9 |
| HSP90AA1 | heat shock protein 90 alpha family class A member 1 | 21 | 14 |
| NME2 | NME/NM23 nucleoside diphosphate kinase 2 | 16 | 16 |
| HIST1H4A | histone cluster 1 H4 family member a | 13 | 284 |
| RPS6 | ribosomal protein S6 | 13 | 5 |
