# Supplementary figures and images for: In Silico Functional Networks Identified in Fish Nucleated Red Blood Cells by Means of Transcriptomic and Proteomic Profiling
Source: Genes (Basel). 2018 Apr 9;9(4):202. doi: 10.3390/genes9040202 (PMC5924544; doi:10.3390/genes9040202)

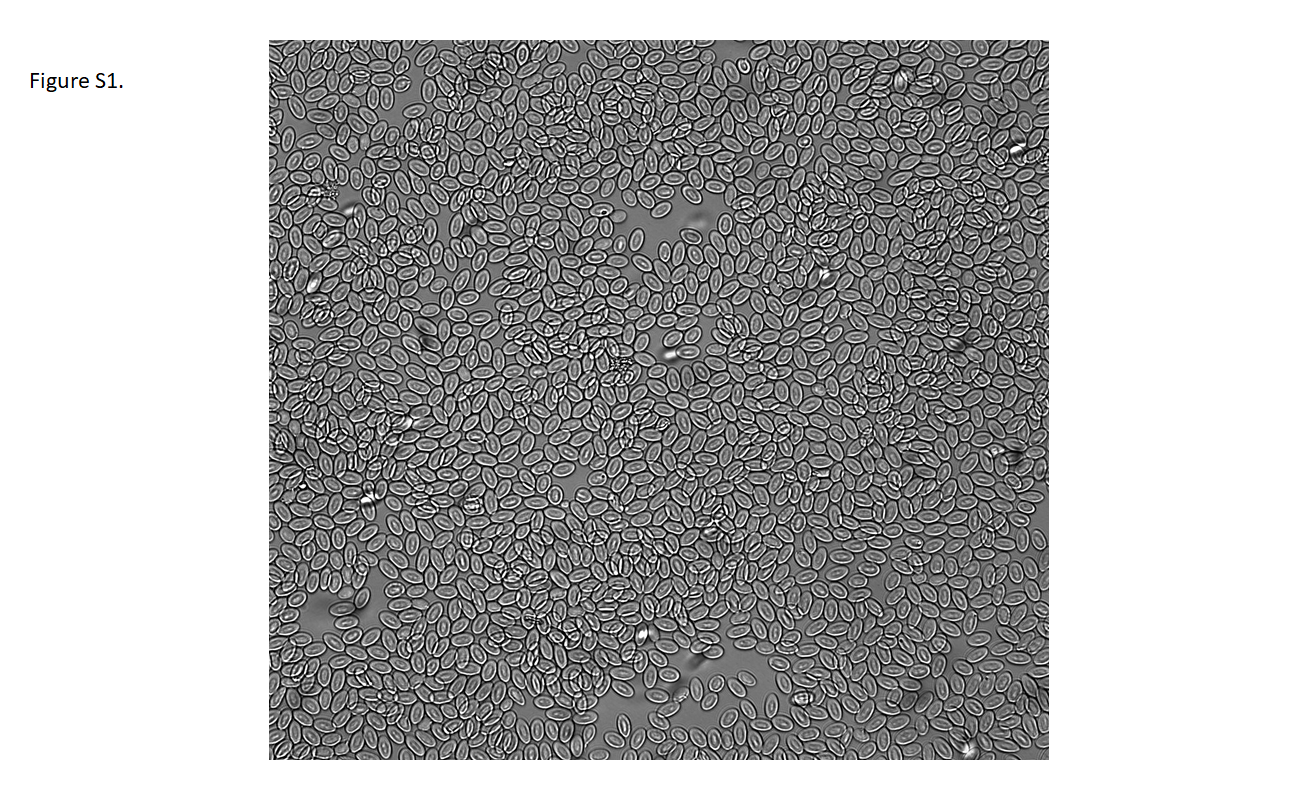

Supplement: Supplementary file 1 [file genes-09-00202-s001.zip › genes-279288_Figure S1_v2.tif]

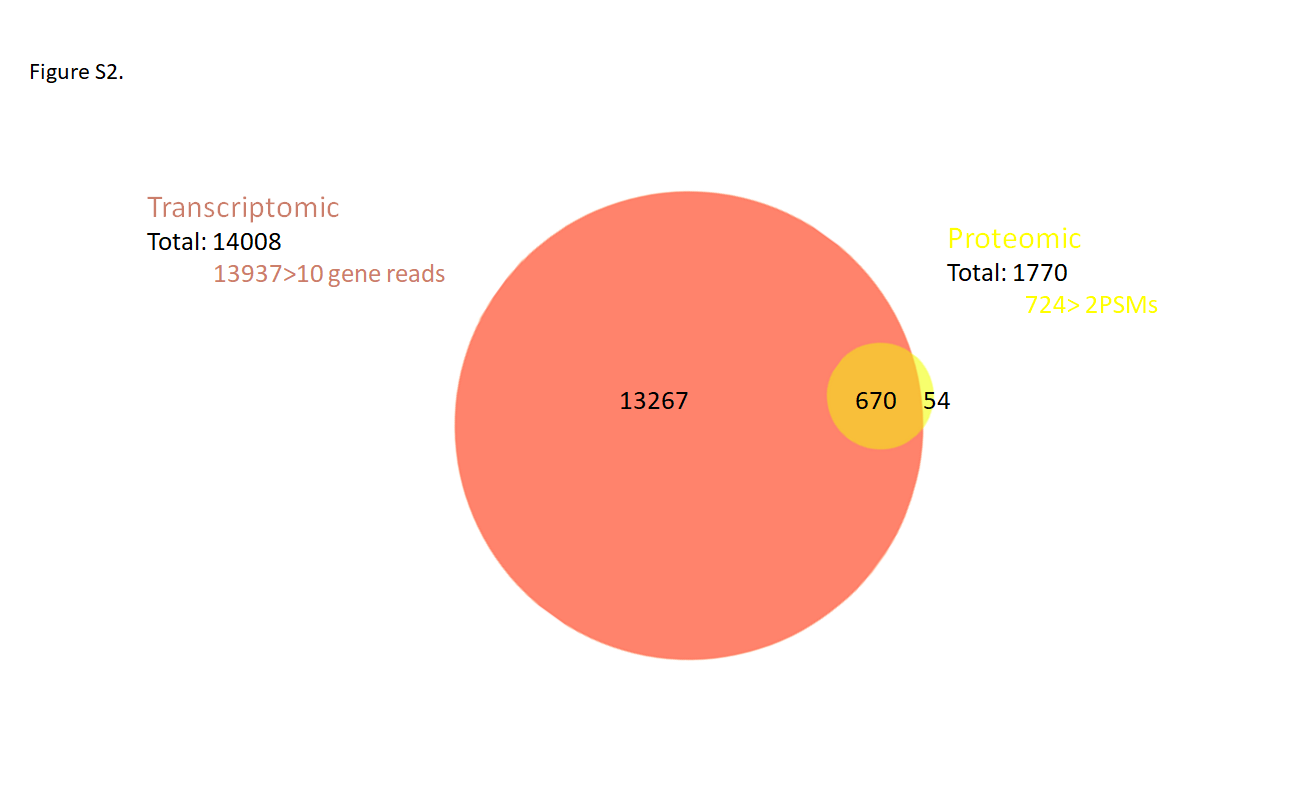

Supplement: Supplementary file 1 [file genes-09-00202-s001.zip › genes-279288_Figure S2_v2.tif]

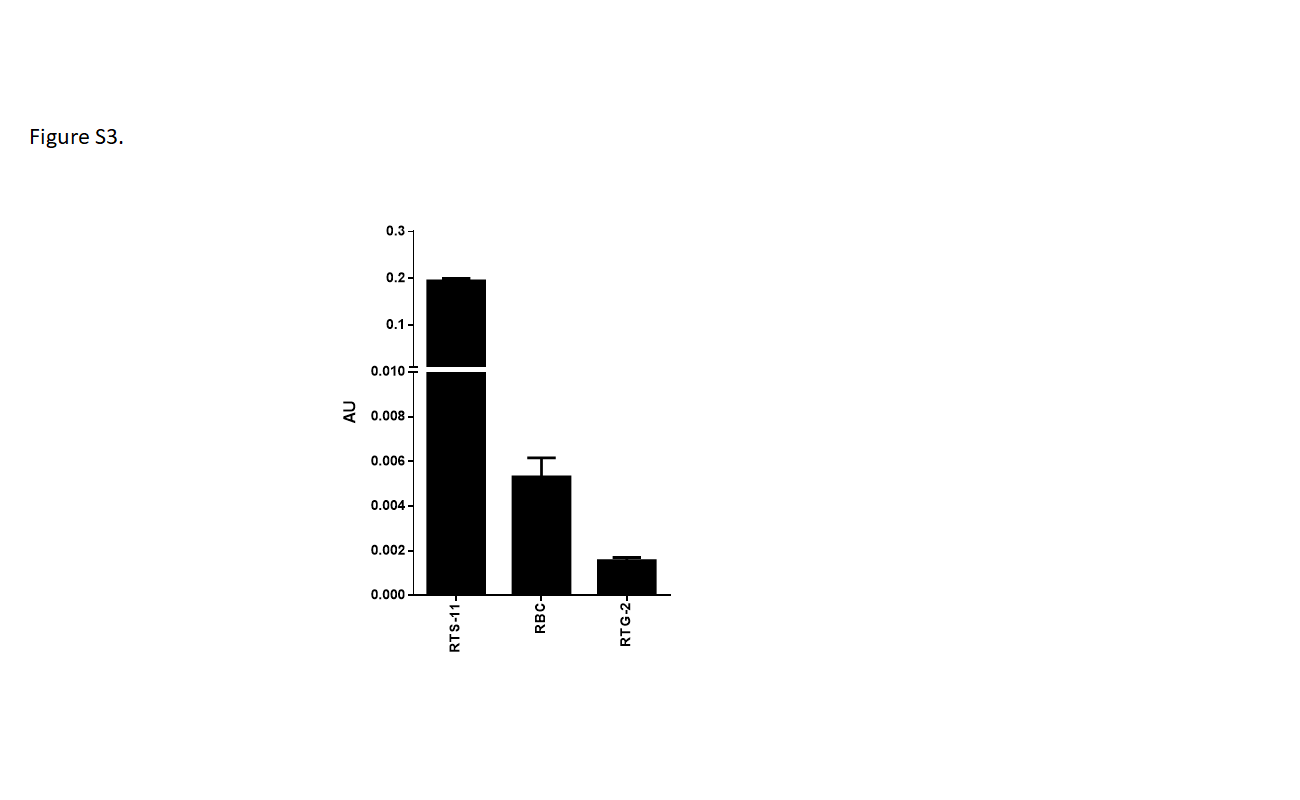

Supplement: Supplementary file 1 [file genes-09-00202-s001.zip › genes-279288_Figure S3_v2.tif]
